# Almonertinib-induced interstitial lung disease in a lung adenocarcinoma patient complicated with interstitial lung abnormality

**DOI:** 10.1186/s12890-023-02367-x

**Published:** 2023-03-08

**Authors:** Qian Zhou, Zhong Hu, Xin Li, Xiaokui Tang

**Affiliations:** 1 Department of Respiratory and Critical Care Medicine, The First Affiliated Hospital of Chongqing Medical University, Chongqing, China; 2 Department of Respiratory and Critical Care Medicine, The First People’s Hospital of Chongqing Liang Jiang New Area, Chongqing, China

**Keywords:** Almonertinib, Interstitial lung disease, EGFR-TKI, Lung adenocarcinoma, Case report

## Abstract

**Background:**

With the use of targeted drugs in lung cancer patients, targeted drug-induced interstitial lung disease (ILD) has attracted more and more attention. The incidence, time, and severity of different targeted drug-induced ILD vary. Almonertinib/HS-10296 is a third-generation epidermal growth factor receptor-tyrosine kinase inhibitor (EGFR-TKI). Post-marketing safety and effectiveness of almonertinib have been confirmed. The reported adverse events of almonertinib were mainly an increase in creatine phosphokinase, aspartate aminotransferase, and alanine aminotransferase, and onset of rash. Almonertinib-induced ILD is rare.

**Case report:**

This paper reported the case of a patient with lung adenocarcinoma complicated with interstitial lung abnormality (ILA). Gene detection showed L858R mutation in exon 21 of the EGFR gene. After operation, almonertinib (110 mg per day) was prescribed. 3 months later, ILD was found by chest CT due to dyspnea.

**Management and outcome:**

Subsequently, almonertinib was stopped. With the administration of intravenous glucocorticoid and oxygen inhalation, the patient's dyspnea was significantly regressed and lung lesions regressed on follow-up chest CT done after discharge.

**Discussion:**

This case suggested that we should pay attention to the existence of ILD/ILA before using targeted drugs. The use of targeted drugs should be more strictly controlled and monitored in patients with previous ILA or ILD. This paper also reviewed the relevant literature on the drug characteristics and summarized the risk factors of ILD caused by EGFR-TKI.

## Background

Lung cancer has a high incidence and mortality. The Global Cancer Observatory predicted that there will be approximately 2.5 million new cases and 2.1 million deaths due to lung cancer by 2025 [1]. Non-small cell lung cancer (NSCLC) accounts for 85% of lung cancer [2]*.* With no specific symptoms in the early stage, a late diagnosis of NSCLC leads to a poor prognosis. Epidermal growth factor receptor (EGFR) mutation plays a carcinogenic role in NSCLC with the most common classical mutations being 19Del and 21L858R [3]. EGFR-tyrosine kinase inhibitor (EGFR-TKI) is the standard first-line treatment for advanced or metastatic NSCLC with EGFR mutation.

Interstitial lung disease (ILD) caused by targeted drugs for lung cancer has attracted much attention. The incidence of ILD and ≥ 3-grade ILD in patients on EGFR-TKI monotherapy was about 1.1–2.2% and 0.6–1%, respectively [4]. Probably due to environmental and genetic polymorphisms, the incidence of ILD in the Japanese population was significantly higher [5, 6]. Previous reports suggested that the incidences of ILD caused by gefitinib, erlotinib, afatinib, dacomitinib, and osimertinib were about 2.6–5.3%, 0.6–1.5%, 0.4–1%, 1.3% and 3%, respectively [3, 4, 7].

The mechanism of ILD induced by EGFR-TKI is not clear. EGFR-TKI can affect the growth and migration of epithelial cells and change the expression of cytokines, resulting in the recruitment of inflammatory cells and subsequent tissue damage; in addition, chronic lung inflammation and interleukin-6 produced by activation of signal TGF-β may be associated with acute lung injury [4]. ILD can sometimes be life-threatening [8].

As a third generation EGFR-TKI, almonertinib has a low affinity for wild-type EGFR and fewer side effects. Almonertinib-induced ILD is rare. Here we reported an ILD related to almonertinib in a patient with NSCLC complicated with interstitial lung abnormality (ILA).

### Case presentation

**Fig. 1:**
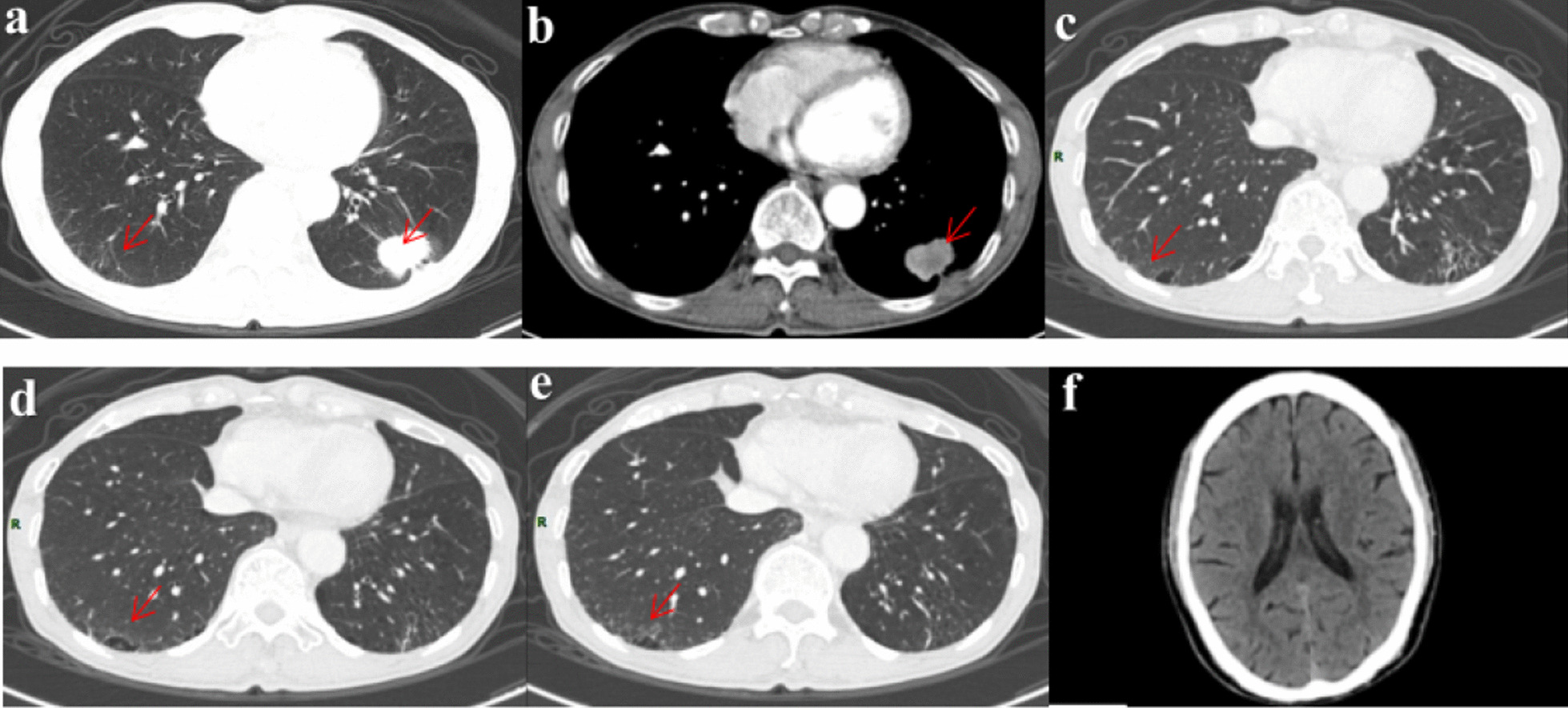
Preoperative CT of the chest and head. **a**–**b** A nodular shadow of 22 mm × 31 mm located in the lower lobe of the left lung with irregularly lobulated shape, spiculation and pleural invasion. **c**–**e** Some reticular opacities and a few ground-glass opacities can be seen in the right lower lung. f No metastasis was found in head CT scan

A 71-year-old man, a former office clerk, was admitted to the First Affiliated Hospital of Chongqing Medical University on December 4, 2020 with a chief complaint of a 22 mm × 31 mm lesion observed on chest computed tomography (CT) since November 26, 2020. As seen in Fig. 1a and b, the lesion was located in the lower lobe of the left lung with irregularly lobulated shape, spiculations, and pleural invasion. This patient had no symptoms in daily life. He had smoked 10 cigarettes a day for 30 years in the past and had quitted smoking for 6 years. He had neither chronic disease nor a family history of cancer.

After admission to the Thoracic Surgery unit, he completed the preoperative workup that showed: carbohydrate antigen 19-9 (CA19-9) at 62.2 U/ml (normal range, 0–27.0 U/ml) and carcinoembryonic antigen (CEA) at 225.1 mg/ml (normal range, 0.2–10.0 mg/ml). No metastasis was found on head CT (Fig. 1f) and whole body bone scan. Contraindications to surgery were excluded by pulmonary function test, electrocardiogram, and echocardiogram. Radical resection of the pulmonary carcinoma and thorax adhesiotomy on thoracoscopy under general anesthesia was performed successfully on December 9, 2020. Intraoperative frozen pathology revealed a cancer. The postoperative pathological examination results revealed an invasive adenocarcinoma of the left lower lung. The proportions of tumor growth patterns of the papillary, acinar, micropapillary, and solid parts were 55%, 30%, 10%, and 5% respectively. No cancer involvement was found in the incisal margin of the bronchus and lung. Metastasis was found in No.5, No.7, No.10, No.11 and No.12 groups of lymph node. No.6, No.8, and No.9 groups of lymph node were not involved. The final diagnosis was invasive adenocarcinoma of the left lower lung classified stage IIIA (T2aN2M0). The result of lung cancer gene detection using paraffin section showed L858R mutation in exon 21 of the EGFR gene along with G12A/V/R/C and G13C mutations in exon 2 of the KRAS gene. Chemotherapy was not given because of the patient's weakness and unwillingness. Before EGFR-TKI selected, we noticed some reticular opacities and a few ground-glass opacities in the right lower lung that affected more than 5% of any lung zone (Fig. 1c–e). Therefore, the patient was considered to have ILA. Considering the low risk of ILD, almonertinib (110 mg per day) was chosen as first-line treatment and started on January 24, 2021.

This patient complained of dyspnea in April 2021 and his activity tolerance decreased significantly. He could only tolerate walking slowly on a flat road. Chest CT (April 23, 2021, Fig. 2) performed at the outpatient showed postoperative changes in the left lung and ILD in the lower lobe of the right lung. No obvious abnormalities were found on whole-body bone imaging. After stopping almonertinib on May 3, 2021, he still had dyspnea. Thus, he was admitted to the first branch of our hospital. As seen in Fig. 3, a repeat chest CT on May 25, 2021 showed an increase in the lesions of ILD in both lungs. The results of his antinuclear antibody, antineutrophil cytoplasmic antibody, myositis antibody, anticyclic citrulline polypeptide antibody, rheumatoid factor, and creatine kinase were negative. Pulmonary function examination showed restrictive ventilation dysfunction and normal diffusion function. His dyspnea did not improve after symptomatic treatment with phlegm removal and anti-asthmatic.

**Fig. 2:**
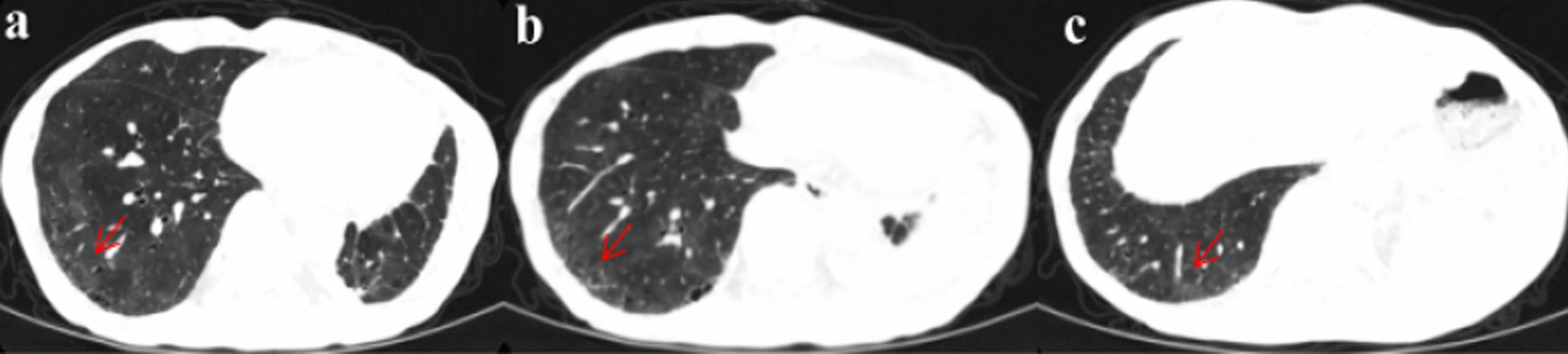
**a**–**c** Chest CT showed interstitial changes in the lower lobe of the right lung after oral administration of almonertinib for 3 months

**Fig. 3:**
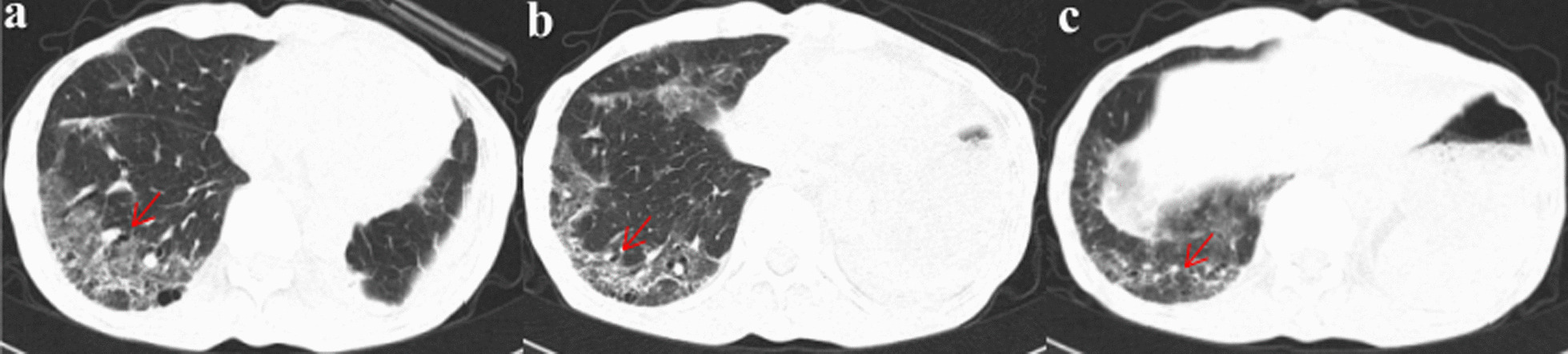
**a**–**c** After 22 days of discontinuation of almonertinib, re-examination of chest CT (May 25, 2021) showed interstitial changes in both lungs, especially in the right lower lung

**Fig. 4:**
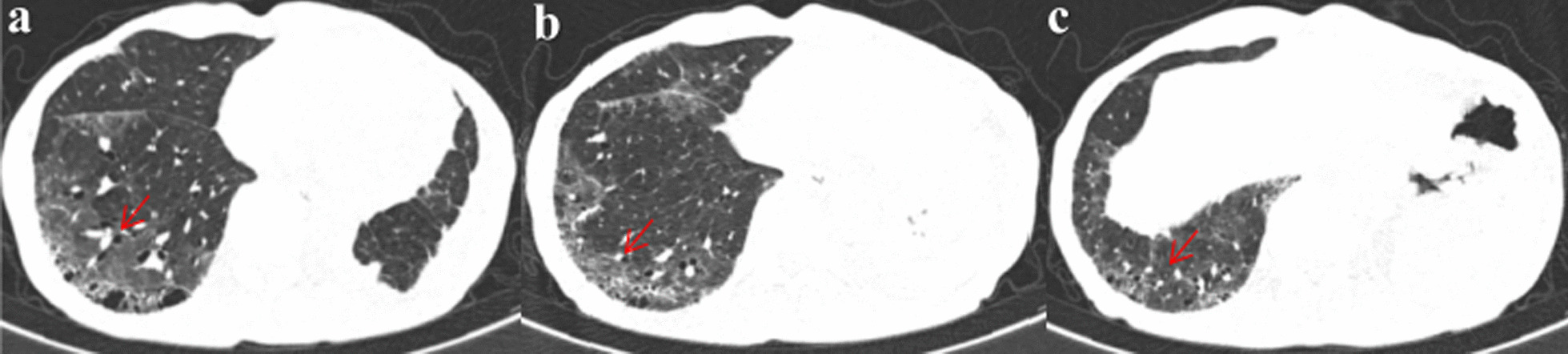
**a**–**c** Five days after treatment with methylprednisolone 40 mg intravenous q12h, chest HRCT (June 8, 2021) showed an improvement in the interstitial inflammation in the lower lobe of the right lung

**Fig. 5:**
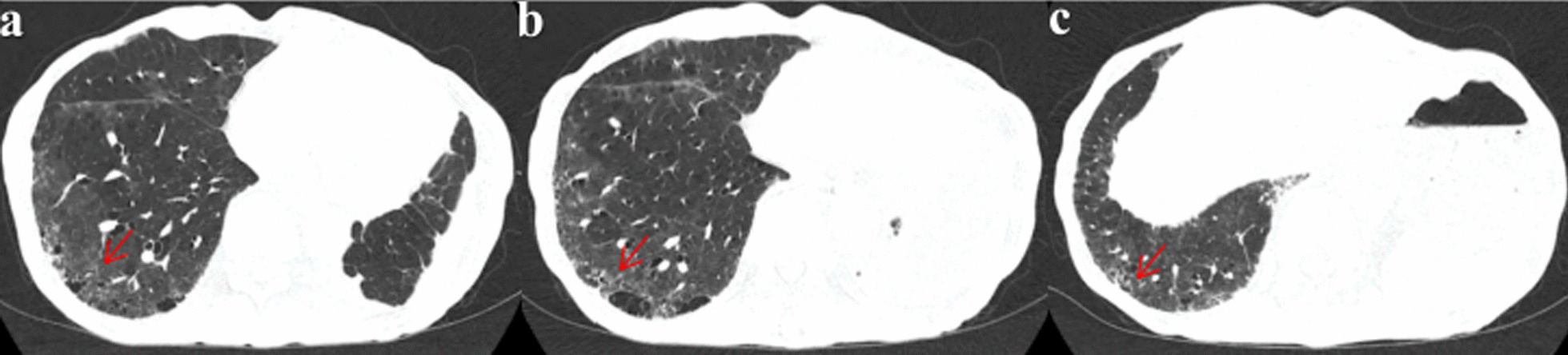
**a**–**c** Thirty-five days after glucocorticoid treatment, chest CT (July 9, 2021) on outpatient follow-up showed a significant reduction in interstitial inflammation than before

For further treatment, the patient was admitted to our hospital on June 3, 2021. Routine tests were requested. Blood gas analysis carried out under a nasal catheter oxygen inhalation of 2 L per minute showed 7.44 for pH, 42 mmHg of arterial partial pressure CO_2_, 85 mmHg of arterial partial pressure of O_2_, and 97% of oxygen saturation. His oxygenation index was 293 mmHg. Hemoglobin was 125 g/L. Infection-related indicators such as white blood cell count, percentage of neutrophils, procalcitonin level, and C-reactive protein were normal. Routine blood tests also revealed normal absolute value and proportion of eosinophils and lymphocytes. There were no abnormalities in liver and kidney function tests, electrolytes, and coagulation function. Cellular immune function monitoring showed CD4 + and CD8 + T cells were 392 per microliter and 143 per microliter respectively. The ratio of CD4 + and CD8 + T cells was 2.74. The specific serum IgM antibodies against influenza A and B virus, respiratory syncytial virus, adenovirus, chlamydia pneumoniae and mycoplasma pneumoniae were negative. The serum (1, 3)-β-D-glucan test and galactomannan detection were negative. Bronchoscopy and bronchoalveolar lavage were not performed due to his respiratory failure and weakness. We organized multidisciplinary discussions. He has never been exposed to dust. He had no history of hair dyeing, keeping pets, or sensitizing substance exposure. He never raised or contacted pigeons. During almonertinib administration, he mainly stayed at home. He did not go to the jungle, park, or other special environments. He did not change his living and eating habits. Amiodarone, immune checkpoint inhibitors, or other drugs that can induce ILD were not used previously. He did not receive radiotherapy or chemotherapy after the operation. The absolute value and proportion of eosinophils in the routine blood were not high. Infection-related indexes were not high. There was no abnormality in connective tissue disease (CTD)-related immune indexes screening. There was no manifestation of heart failure. According to imaging characteristics and negative whole body bone imaging, there was insufficient evidence of tumor recurrence. Based on the above analysis, we excluded eosinophilic pneumonia, hypersensitivity pneumonitis, pulmonary infection, CTD-ILD, heart failure, tumor recurrence, and other drugs-induced lung disorders. Considering the onset of ILD 3 months after taking almonertinib, almonertinib-induced ILD was evoked. Acetylcysteine 0.6 g q8h was used for antioxidation. Bailing capsule 1 g q8h was given as adjuvant treatment for ILD. On June 4, 2021, methylprednisolone 40 mg q12h was administered intravenously, supplemented by calcium tablet and stomach protection drugs. The patient's respiratory condition gradually improved on this treatment. On June 8, 2021, high resolution lung CT (Fig. 4) showed an improvement in the interstitial inflammation of the lower lobe of the right lung. On June 10, 2021, methylprednisolone dosage was reduced to 40 mg per day. On June 15, 2021, routine blood, liver, and kidney function tests and electrolyte were tested and showed no obvious abnormality. The patient was given oral prednisone tablets 40 mg per day and he was followed up regularly in the outpatient department. Prednisone dosage was gradually reduced. On July 9, 2021, repeat chest CT (Fig. 5) showed a significant reduction in the interstitial inflammation. So far, the patient's respiratory condition is stable. The patient tolerated this therapeutic schedule well with no other side effect.

## Discussion

EGFR-TKI is the standard first-line treatment for advanced or metastatic NSCLC in patients with EGFR mutation. EGFR-TKI can block signal transduction in tumor cells by competitively inhibiting TK phosphorylation, tumor growth, and metastasis. T790M mutation is characterized by methionine replacing threonine at position 790 of EGFR. This can hinder the binding of EGFR-TKI to EGFR, resulting in increased affinity between EGFR and adenosine triphosphate [1]. Therefore, most patients develop drug resistance after using the first or second generation EGFR-TKI for 9–14 months [9]. The third generation EGFR-TKI can irreversibly bind to EGFR ATP binding site and EGFR T790M resistance mutation to inhibit phosphorylation of mutant EGFR and block the downstream signal transduction. As a result, NSCLC cells proliferation is inhibited [7, 10].

Almonertinib, a new third generation EGFR-TKI, takes aminopyrimidine as the parent nucleus and retains the Michael addition receptor. Almonertinib and its metabolites have weak inhibition on wild-type EGFR and have less common side effects [9]. The lipophilicity of cyclopropyl ensures good blood–brain barrier penetration [7, 11]. Phase I and II studies showed that almonertinib had strong anticancer activity against advanced and metastatic NSCLC with sensitive EGFR or T790M mutations [2, 9]. In the phase III study, the median progression-free survival was 19.3 months [12]. On December 16, 2021, almonertinib was approved by the National Medical Products Administration as first-line treatment of locally advanced or metastatic NSCLC with 19Del and 21L858R mutation. At the European Lung Cancer Congress (ELCC) in 2022, Hu Jian's research proved for the first time that almonertinib had significant efficacy and good safety as postoperative adjuvant treatment of stage I-III NSCLC with EGFR mutation [13].

The main adverse events of almonertinib are rash and elevation of creatine phosphokinase, aspartate aminotransferase, and alanine aminotransferase, and ILD was rare. In the phase I study, a treatment-related grade 4 ILD was also recorded in the cohort receiving 260 mg [9]. There was no ILD reported in APOLLO study (phase II study) [2, 7, 10]. In the AENEAS study (phase III study), 2 cases of ILD (which was grade 2) were observed [12]. In 2020, Ting Jiang and colleagues reported a case of almonertinib-induced ILD [3]. Longqiu Wu reported a case of successful treatment of EGFR T790M-mutant NSCLC with almonertinib after osimertinib-induced ILD occurred [7]. As mentioned earlier, the third generation EGFR-TKI has low selectivity for wild-type EGFR, which may be an explanation for the low number of ILD cases. However, factors such as sample size and follow-up time should still be considered.

Here we reported a lung adenocarcinoma patient complicated with ILA. According to L858R mutation in exon 21, we selected almonertinib as treatment. Close observation was given to him in follow up. Dyspnea occurred 3 months later. Chest CT showed ILD. Before almonertinib-induced ILD was considered, our ILD team carefully excluded eosinophilic pneumonia, hypersensitivity pneumonitis, pulmonary infection, CTD-ILD, heart failure, tumor recurrence, and other drugs-induced lung disorders. His symptoms improved gradually after using methylprednisolone. Regression of ILD lesions on repeat chest CT further suggested the right diagnosis and good curative effect. The Naranjo algorithm score was 9. Some serological markers such as KL-6 and SP-D have a certain role in predicting and evaluating the curative effect of ILD. However, these are not reimbursed by the medical insurance in China and the patient refused to do them. If these markers could be performed in this patient, more basis for the use of these markers in ILD could be provided. This is the first case report of almonertinib-induced ILD in patients with ILA. However, the safety of almonertinib in patients with ILA or ILD needs more research because this was a single case study.

ILA, a radiological definition, refers to non-dependent abnormalities such as ground-glass or reticular abnormalities, architectural distortion, traction bronchiectasis, honeycombing and non-emphysematous cysts affecting more than 5% of any lung area in patients without clinical suspicion of ILD. This occurs in 4–9% of smokers and 2–7% of non-smokers [14]. In the lung cancer screening population, the prevalence of ILA was about 9.7% [15]. ILA is associated with therapeutic toxicity and mortality of lung cancer. Washko reported an effective and efficient scoring method for ILA on chest CT using a four-point system. A higher ILA score is associated with a shorter overall survival. Thus, ILA may be a marker of a shorter survival time in advanced NSCLC [16]. For lung cancer patients presenting with ILA, we should choose the appropriate treatment scheme according to the individual situation rather than absolutely excluding the optional scheme in view of the possible risks. On the contrary, we need to make efforts to carefully control, closely monitor, and timely adjust treatment according to the situation.

Up to now, most large-scale clinical trials on lung cancer did not include patients with ILD. There have been studies on the risk factors of ILD caused by lung cancer targeted drugs. These nine clinical studies, as shown in Table 1, were all carried out in Japan. The consensus was that concurrent or previous ILD was an important risk factor of targeted drugs-induced ILD. Sometimes this situation was described as acute exacerbation of previous ILD. Other risk factors of targeted drugs-induced ILD included age, gender, smoking history, high performance status score, previous radiotherapy history, previous use of nivolumab, comorbidities including heart disease and chronic obstructive pulmonary disease, and a lower residual normal lung tissue. Studies on gefitinib and erlotinib occupied the majority. As shown in Table 1, the onset time range of ILD was more than 10 days to several weeks. In the overall study population of Table 1, the incidence of targeted drugs-induced ILD, targeted drugs-induced ILD above grade 3, and mortality were 1.0–6.5%, 0–3.6%, and 0–75.0%, respectively. In patients with concurrent or previous ILD, the incidence of targeted drugs-induced ILD and mortality were 8.8–30.0% and 0–83.3%, respectively. Overall, patients with concurrent or previous ILD are more likely to develop targeted drugs-induced ILD and have a higher mortality. Further studies are needed to confirm this finding. In the population with targeted drugs-induced ILD, the proportion of acute interstitial pneumonia (AIP)/diffuse alveolar damage (DAD) on chest CT was 0–62.5%. Specifically, the AIP/DAD proportion of gefitinib, crizotinib, erlotinib, and osimertinib were 40.0–62.5%, 27.4%, 0–14.7%, and 11.3%, respectively. Endo analyzed the clinical data of acute ILD caused by gefitinib and found that AIP had a prevalence of 23.5% with mortality of 75.0% [4]. Patients with CT pattern of AIP/DAD may have a worse prognosis [6]. The differences between targeted drugs-induced ILD need further exploration.

**Table 1:** Clinical studies on risk factors of ILD caused by lung cancer targeted therapy

No	Author, publication time	EGFR-TKIs	Number of study people	Onset time of ILD	Incidence/mortality of ILD		Incidence of ILD (≥ grade 3)	AIP/DAD
					General population	Patients with concurrent or previous ILD		
1 [17]	Katsuyuki Hotta, (2005)	Gefitinib	330	Median time of onset was 22d	15 (4.5%)/8 (53.3%)	6 (30.0%)/5 (83.3%)	12 (3.6%)	6 (40.0%)
2 [18]	Masahiko Ando, (2006)	Gefitinib	1976	median time of onset was 31d	70 (3.5%)/31 (44.3%)	5 (13.9%)/–	–	–
3 [19]	Shoji Kudoh, (2008)	Gefitinib	1901	Onset within 28d was most common	79 (4.2%)/25 (31.6%)	–	–	–
4 [20]	Katsuyuki Hotta, (2010)	Erlotinib (group A) vs gefitinib (group B)	539	Median time of onset was 13d	Group A: 2 (1.0%) /0 (0%); Group B: 8 (2.4%) /6 (75%)	Group A: 1 (20.0%)/0 (0%); Group B: 5 (25.0%)/4 (80.0%)	Group A: 0 (0%); Group B: 8 (2.4%)	Group A: 0 (0%) Group B: 5 (62.5%)
5 [21]	Shigeo Kawase, (2011)	Gefitinib or erlotinib	341	Median time of onset was 19d	20(5.9%)/9(45.0%)	9 (8.8%)/6 (66.7%)	–	5(25%)
6 [22]	Akihiko Gemma, (2014)	Erlotinib	9909	Median time of onset was 28d	429 (4.3%)/153 (35.7%)	–	257(2.6%)	63(14.7%)
7 [23]	Takeshi Johkoh, (2014)	Erlotinib	627	80.0% occurred within 28 days	19 (3.0%)/6 (31.6%)	5 (10.6%)/0 (0%)	9 (1.4%)	–
8 [24]	Akihiko Gemma, (2018)	Crizotinib	2028	41.9% occurred within 4 weeks; 69.2% occurred within 8 weeks	117 (5.8%)/22 (18.8%)	15 (23.1%)/–	70 (3.5%)	32 (27.4%)
9 [25]	Akihiko Gemma, (2020)	Osimertinib	3578	Median time of onset was 63d	231 (6.5%)/29 (12.6%)	20 (19.23%)/–	104 (2.9%)	26 (11.3%)

No	Author, publication time	Targeted drugs	Risk factors of ILD
1 [17]	Katsuyuki Hotta, (2005)	Gefitinib	Previous pulmonary fibrosis; PS of 3–4 points; radiotherapy history
2 [18]	Masahiko Ando, (2006)	Gefitinib	Concurrent ILD; male; smoking history
3 [19]	Shoji Kudoh, (2008)	Gefitinib	Preexisting ILD; PS (> 2 points); older age (> 55 years old); smoking history; short duration since NSCLC diagnosis (< 6 months); reduced extent of normal lung on CT scan (< 50%); concurrent cardiac disease
4 [20]	Katsuyuki Hotta, (2010)	Erlotinib (group A) vs gefitinib (group B)	Previous pulmonary fibrosis; PS of 3–4 points
5 [21]	Shigeo Kawase, (2011)	Gefitinib or erlotinib	Previous pulmonary fibrosis
6 [22]	Akihiko Gemma, (2014)	Erlotinib	Concurrent or previous ILD; emphysema or COPD; Lung infection; smoking history; time from cancer diagnosis to treatment (< 360 days)
7 [23]	Takeshi Johkoh, (2014)	Erlotinib	Previous ILD; reduced extent of normal lung on CT scan (< 50%)
8 [24]	Akihiko Gemma, (2018)	Crizotinib	Concurrent or previous ILD; older age; PS of 2–4 points; smoking history; concurrent pleural effusion
9 [25]	Akihiko Gemma, (2020)	Osimertinib	Concurrent or previous ILD; history of using nivolumab

In conclusion, we need to monitor for the occurrence of ILD secondary to almonertinib. ILD/ILA should be checked before using EGFR-TKIs. Lung cancer patients with ILD or ILA in the past need more rigorous monitoring and follow-up in the process of targeted drug therapy.

## Data Availability

The clinical data reported in this article was extracted from electronic medical record system along with picture archiving and communication system (PACS) of our hospital. Follow up data was obtained from outpatient records and telephone calls. For further data, please contact the corresponding author.

## Abbreviations

ILD: Interstitial lung disease
EGFR-TKI: Epidermal growth factor receptor-tyrosine kinase inhibitor
ILA: Interstitial lung abnormality
NSCLC: Non small cell lung cancer
CA19-9: Carbohydrate antigen 19-9
CEA: Carcinoembryonic antigen
BAL: Bronchoscopy and bronchoalveolar lavage
ASCO: American society of clinical oncology
ELCC: European lung cancer congress
CTD: Connective tissue disease
PS: Performance status
COPD: Chronic obstructive pulmonary disease
AIP: Acute interstitial pneumonia
DAD: Diffuse alveolar damage
PACS: Picture archiving and communication system
